# High performance microbiological transformation of L-tyrosine to L-dopa by *Yarrowia lipolytica *NRRL-143

**DOI:** 10.1186/1472-6750-7-50

**Published:** 2007-08-16

**Authors:** Sikander Ali, Jeffry L Shultz, Ikram-ul-Haq

**Affiliations:** 1 Institute of Industrial Biotechnology (IIB), GC University Lahore, Katchehry Road, Lahore-54000, Pakistan; 2 USDA-ARS, Crop Genetics and Production Research Unit, Stoneville, MS 38776, USA

## Abstract

**Background:**

The 3,4-dihydroxy phenyl L-alanine (L-dopa) is a drug of choice for Parkinson's disease, controlling changes in energy metabolism enzymes of the myocardium following neurogenic injury. *Aspergillus oryzae *is commonly used for L-dopa production; however, potential improvements in ease of handling, growth rate and environmental impact have led to an interest in exploiting alternative yeasts. The two important elements required for L-dopa production are intracellular tyrosinases (thus pre-grown yeast cells are required for the transformation of L-tyrosine to L-dopa) and L-ascorbate, which acts as a reducing agent.

**Results:**

Pre-grown cells of *Yarrowia lipolytica *NRRL-143 were used for the microbiological transformation of L-tyrosine to L-dopa. Different diatomite concentrations (0.5–3.0 mg/ml) were added to the acidic (pH 3.5) reaction mixture. Maximum L-dopa biosynthesis (2.96 mg/ml L-dopa from 2.68 mg/ml L-tyrosine) was obtained when 2.0 mg/ml diatomite was added 15 min after the start of the reaction. After optimizing reaction time (30 min), and yeast cell concentration (2.5 mg/ml), an overall 12.5 fold higher L-dopa production rate was observed when compared to the control. Significant enhancements in Y_p/s_, Q_s _and q_s _over the control were observed.

**Conclusion:**

Diatomite (2.0 mg/ml) addition 15 min after reaction commencement improved microbiological transformation of L-tyrosine to L-dopa (3.48 mg/ml; p ≤ 0.05) by *Y. lipolytica *NRRL-143. A 35% higher substrate conversion rate was achieved when compared to the control.

## Background

The yeast *Yarrowia lipolytica *is a hemiascomycete and represents a homogeneous phylogenetic group with physiological and ecological diversity [1]. It is a non-conventional yeast, often used in research and isdistantly related to *Candida glabrata*, *Kluyveromyces lactis *and *Debaryomyces hansenii*. Strains of *Y. lipolytica *can produce significant amounts of intra- or extra-cellular metabolites including vitamins, lipases, storage lipids, citric acid and pyruvic acid and can be used for biodegradation of various wastes (e.g., olive-mill waters and raw glycerol) [2-6]. The 3,4-dihydroxy phenyl L-alanine (L-dopa) is a drug used for Parkinson's disease, and is capable of changing the enzymes of energy metabolism of myocardium following neurogenic injury. The process of bioconversion of L-tyrosine to L-dopa in microorganisms is generally slow, but is accelerated by a small amount of L-dopa in the broth [7]. L-dopa has also been produced with *Erwinia herbicola *cells carrying a mutant transcriptional regulator TyrR from pyrocatechol and DL-serine [8,9]. It can also be produced using L-tyrosine as a substrate, tyrosinase as a biocatalyst and L-ascorbate as the reducing agent [10,11]. The general reaction is:



Tyrosinases (monophenol, o-diphenol:oxygen oxidoreductase, EC 1.14.18.1) belong to a larger group of type-3 copper proteins, which include catecholoxidases and oxygen-carrier haemocyanins [12]. Tyrosinases are involved in the melanin pathway and are responsible for the first steps of melanin synthesis from L-tyrosine, leading to the formation of L-dopaquinone and L-dopachrome [13]. Tyrosinases catalyse the o-hydroxylation of monophenols (cresolase activity or "monophenolase") and the ensuing oxidation of molecular oxygen. Subsequently, the o-quinones undergo non-enzymatic reactions with various nucleophiles, producing intermediates [14]. The immobilization of tyrosinases on solid supports can increase enzyme stability [15-19], protect tyrosinase from inactivation by reaction with quinones, (preserving them from proteolysis) [20], improve thermal stability of fungal tyrosinases [21], and increase activity in comparison to soluble enzymes [22].

Diatomite (2:1 clay mineral) is a naturally occurring, soft, chalk-like sedimentary rock that is easily crumbled into a fine, off-white powder which has K^+ ^in the interlayer. This powder has an abrasive feeling similar to pumice and is light-weight due to its porosity. By adding diatomite into the reaction, an increased substrate uptake and enzyme production rate with concomitant L-dopa production could result. We have previously reported the effect of cresoquinone and vermiculite on the microbial transformation of L-tyrosine to L-dopa by *Aspergillus oryzae *[23,24]. In the present study, different concentrations of diatomite were added into the reaction mixture to achieve a high performance transformation of L-tyrosine to L-dopa.

*A. oryzae *is an organism typically used for L-dopa production. The easy handling, rapid growth rate and environmentally friendly nature of alternative yeasts such as *Y. lipolytica *have created an interest in their use for fermentation. Because tyrosinases are intracellular enzymes, pre-grown cells harvested from fermented broth were used for the microbiological transformation of L-tyrosine to L-dopa.

## Results and discussion

**Figure 1:**
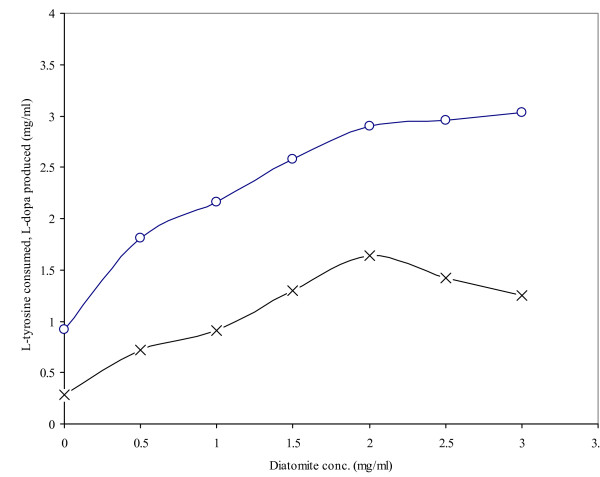
The effect of different diatomite concentrations on L-dopa production by *Y. lipolytica *NRRL-143 (L-tyrosine consumed -∘-, L-Dopa produced -×-). A total of 3.5 mg/ml L-tyrosine, 3.0 mg/ml cell biomass and varying diatomite amounts were added at the start of the biochemical reaction. The total reaction time was 50 min at 50°C.

The production of L-dopa is largely dependent on the addition of specific additives and minerals to the reaction mixture. The inductive effect of diatomite on the transformation of L-tyrosine to L-dopa by *Yarrowia lipolytica *NRRL-143 was investigated (Figure 1). The concentration of diatomite added at the start of biochemical reaction ranged from 0.5–3.0 mg/ml, along with 3.5 mg/ml L-tyrosine. A biomass concentration of 3.0 mg/ml was used as a source of intracellular enzyme tyrosinase in a 50 min reaction. The highest production of L-dopa (1.64 mg/ml produced with 2.90 mg/ml consumption of L-tyrosine) was observed with 2.0 mg/ml diatomite. L-dopa production fell while substrate consumption continued to rise, probably due to catecholase activity causing L-dopa to be used for quinone production, since ascorbic acid (which inhibits this activity) was not being replaced in the system. In some enzyme systems, disaccharides or higher molecular weight substrates have been found to be the best supporters of intracellular enzymes [25,26]. It was hypothesized that tyrosinase, a constitutive enzyme, was altered with respect to production of L-dopa in the presence of added diatomite.

**Figure 2:**
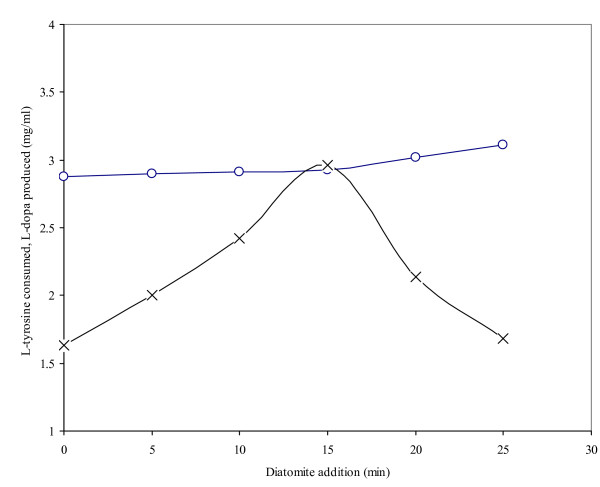
The effect of time of addition of diatomite on L-dopa production by *Y. lipolytica *NRRL-143 (L-tyrosine consumed -∘-, L-Dopa produced -×-). A total of 2.0 mg/ml diatomite was added to 3.5 mg/ml L-tyrosine and 3.0 mg/ml cell biomass. The total reaction time was 50 min at 50°C.

The effects of delayed diatomite addition (2.0 mg/ml; 0, 5, 10, 15, 20, 25 min) into the *Y. lipolytica *NRRL-143 reaction were also investigated (Figure 2). Reactions were performed aerobically with 3.0 mg/ml cell biomass and 3.5 mg/ml L-tyrosine for 50 min. Production of L-dopa increased from 5 to 15 min after the addition of diatomite; a significant decrease of L-dopa (1.68–2.14 mg/ml) was noticed 20–25 min after the addition. Maximum L-dopa (2.96 mg/ml) was obtained 15 min after the addition of diatomite into the reaction mixture, with concomitant tyrosine consumption of 2.94 mg/ml, a 35% increase when compared to the control which is highly significant (p ≤ 0.05). The L-tyrosine substrate has binding affinity with diatomite, which induces tyrosinase secretion, improves its availability and ultimately leads to an increased L-dopa production rate [7,11,13,24]. In our experiment, the addition of diatomite 15 min after reaction commencement was identified as optimal, increasing production of L-dopa, substrate utilization and time of reaction. However, L-dopa production dropped (1.68 mg/ml with 3.14 mg/ml L-tyrosine consumption) when diatomite was added 25 min after the start of reaction, probably due to conversion of unstable L-dopa to dopamine, melanin and other pigmented products [10,13] after a reduced availability of the enzyme.

The consumption of L-tyrosine, however, continued to increase despite the time of diatomite addition. The tyrosinase active center is comprised of dinuclear copper, coordinated with histidine residues, chelating substances or substances that are associated with this metal (as are quinones) which are irreversible inhibitors and/or inactivators of this enzyme [12]. The addition of diatomaceous earth may remove these inhibitors and/or inactivators by active absorption. The absorption of inhibitors increased the enzyme activity of tyrosinases, β-carboxylases and tyrosine hydroxylases which was important for the catabolism of L-tyrosine to L-dopa under controlled conditions. Our data are both substantiated [25] and in contrast to previous reports [26] in which the production of L-dopa was achieved in minimal medium without additive supplementation (pH 7.0). Previous research efforts to produce L-dopa by the addition of 0.16 μg vermiculite during the reaction obtained 0.39–0.54 mg/ml of the desired product [27].

**Figure 3:**
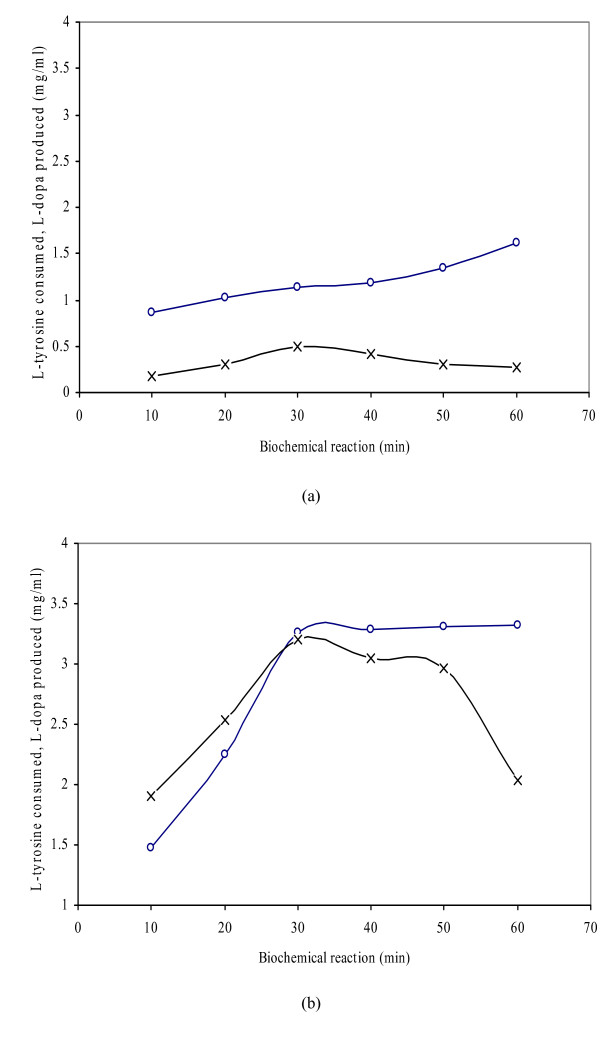
Time course of L-dopa production and L-tyrosine consumption by *Y. lipolytica *NRRL-143 (L-tyrosine consumed -∘-, L-Dopa produced -×-). a. Control (3.5 mg/ml L-tyrosine and 3.0 mg/ml cell biomass) b. Test (2.0 mg/ml diatomite added 15 min after the start of reaction to 3.5 mg/ml L-tyrosine and 3.0 mg/ml cell biomass). The total reaction time was 50 min at 50°C.

The time course of L-dopa production and L-tyrosine consumption was carried out at different incubation periods (10–60 min) using a hotplate with magnetic stirrers (Figure 3). The control gave a maximum of 0.50 mg/ml L-dopa with 1.14 mg/ml consumption of L-tyrosine. The maximum conversion rate (3.20 mg/ml L-dopa with 3.26 mg/ml tyrosine consumption) was obtained with 2.0 mg/ml diatomite added 15 min after the start of reaction, producing a 72% higher yield of L-dopa compared to the control. The L-dopa production from this time course differed significantly (p ≤ 0.05) with the results at all other incubation periods. It is clear that up to 30 min, cresolase activity predominated and, given the non-replacement of ascorbic acid, the overriding activity was catecholase, which consumed the L-tyrosine substrate without a corresponding production of L-dopa. After 40–60 min of incubation, the production of L-dopa and the consumption of L-tyrosine decreased gradually in the control and test reactions. This reduction might be because the L-dopa and residual L-tyrosine were changed into other metabolites such as dopamine, melanin and eventually melanosine. Another study [25] achieved 0.12 mg/ml of L-dopa, 90 min after the biochemical reaction. The present finding of 3.20 mg/ml L-dopa after 30 min of incubation is a major improvement. In the present study, dopamine and melanin were also produced, but their highest production was 0.014 and 0.01 mg/ml/h.

**Figure 4:**
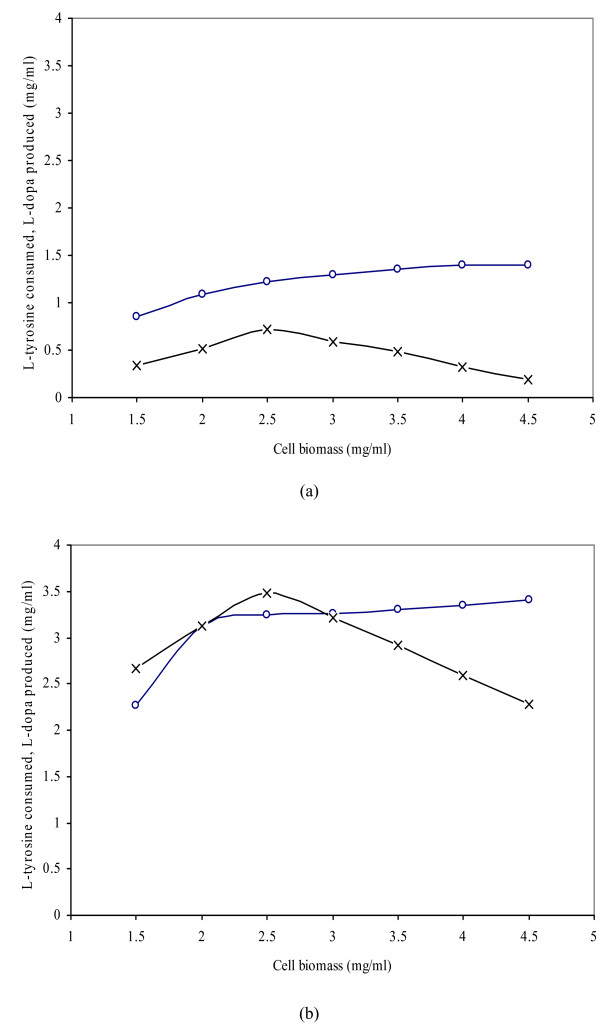
The effect of various levels of cell biomass (*Y. lipolytica *NRRL-143) on L-dopa production (L-tyrosine consumed -∘-, L-Dopa produced -×-). a. Control (3.5 mg/ml L-tyrosine only). b. Test (3.5 mg/ml L-tyrosine and 2.0 mg/ml diatomite added 15 min after the start of reaction), The total reaction time was 50 min at 50°C.

Conversion of L-tyrosine to L-dopa is an enzyme catalyzed reaction. Figure 4 shows the effect of the addition of different concentrations of drenched cell biomass (1.5, 2.0, 2.5, 3.0, 3.5, 4.0, 4.5 mg/ml) on the production of L-dopa from L-tyrosine in the reaction mixture. The best results (3.48 mg/ml L-dopa with 3.25 mg/ml L-tyrosine consumption) were obtained using 2.5 mg/ml wet weight yeast cells, leading to 10 fold higher productivity when compared to the control (0.72 mg/ml L-dopa with 1.22 mg/ml L-tyrosine consumption). At this concentration (2.5 mg/ml), most of the added tyrosine was converted to L-dopa as indicated by the small amount of residual substrate (0.25 mg/ml), which is highly significant (p ≤ 0.05). In the present investigation, the increased cell biomass enhanced enzymatic activity (1.55 U/mg tyrosinase). However, increasing the cellular concentration beyond the optimal led to a sharp decrease in activity, probably due to increased cell concentration (proportional to enzyme concentration) and the maintenance of a constant concentration of an inhibitor of catecholase activity (ascorbic acid). This product is the substrate for the second reaction catalyzed by this enzyme (catecholase activity) which leads to the formation of quinones from L-dopa. Only an excessive amount of ascorbic acid continually replaced throughout the reaction might stop this second activity from taking place, leading to the formation of quinones that are also suicide inactivators of this enzyme. Previous research [28] pointed out that tyrosinase activity is directly related to the concentration of cells or mycelia in the reaction mixture in slightly acidic to neutral reaction conditions. Copper atoms found at the active site of tyrosinase are an essential requirement for catalytic activity. Agents such as carbon monoxide or toxins indirectly inhibit tyrosinase activity by chelating copper and abrogating its ability to bind oxygen. Previous research [8,12,13] has shown that tyrosine phenol lyase (*tpl*) is only synthesized under L-tyrosine-induced conditions. The addition of L-tyrosine to the medium was found unavoidable when preparing cells (the enzyme source), but severely impeded preparation of pure L-dopa [24].

A comparison of production parameters for the effect of diatomite addition on bioconversion of L-tyrosine to L-dopa is shown in Table 1. An overall 12.5 fold increase in L-dopa production (with 4.06 mg/ml proteins) was achieved at the optimal level of added diatomite when compared to the control. The optimal pH of the control reaction without added diatomite was 3.5, however, the test reaction with added diatomite was proficient at a pH range of 2.5–4.0, indicating the enzyme remained active despite the change in reaction pH. The Y_p/s _value (with 2.0 mg/ml diatomite added 15 min after the start of reaction) was significantly improved over the control. Maximum substrate consumption (Q_s_) in terms of volumetric rate was marginally different during bioconversion between the control and test reactions, indicating maximum enzyme activity at this level of diatomite addition. The increase of q_s _(i.e., specific substrate consumption rate) with diatomite addition was highly significant (p ≤ 0.05). In the present study, the optimal values of all kinetic parameters (Y_p/s_, Q_s _and q_s_) were several-fold improved over those reported from *Aspergillus *or *Cellulomonas *spp. [7,10,28].

**Table 1:** Comparison of parameters for L-dopa production by *Y. lipolytica *NRRL-143

Production parameters*	Control	Test**
Proteins (mg/ml)	0.34	4.06
Y_p/s_	0.590	1.071
Q_s_	0.073	0.329
q_s_	0.002	0.011
Optimal pH^$^	3.5	2.5–4.0

Max. L-dopa production (mg/ml)	0.28	3.48

Level of significance <p>*^$^	-	HS

*Kinetic parameters: Y_p/s _= mg L-dopa produced/mg substrate consumed, Q_s _= mg substrate consumed/ml/h, q_s _= mg substrate consumed/mg cells/h.

**2.0 mg/ml diatomite added 15 min after the start of reaction.

^$^Acetate buffer

*^$^<p> is for significance level (≈0.05) on the basis of probability. HS denotes that the values are highly significant.

## Conclusion

In the present studies, *Yarrowia lipolytica *strain NRRL-143 was exploited for L-dopa production. The addition of 2.0 mg/ml diatomite (2:1 clay mineral) markedly improved the microbiological transformation of L-tyrosine to L-dopa. Diatomite addition 15 min after the start of reaction produced a 35% higher substrate conversion rate compared to the control (p ≤ 0.05). A biomass concentration of 2.5 mg/ml and reaction time of 30 min were also optimized. Because production of L-dopa is a high cost, low yield process, scaled up studies are a pre-requisite for commercial exploitation.

## Methods

### Microorganism and growth conditions

*Yarrowia lipolytica *strain NRRL-143 was grown on yeast extract agar slants (pH 5.4) and stored in a cold-cabinet (Model: 154P, Sanyo, Tokyo, Japan) at 4°C. Two hundred milliliters of cultivation medium containing (% w/v); glucose (2.0, polypeptone (1.0), NH_4_Cl 0.3, KH_2_PO_4 _(0.3), MgSO_4_·7H_2_O (0.02), yeast extract (1.0) (pH 5.5) were taken into individual 1.0 L Erlenmeyer flasks. The medium was autoclaved at 15 psi (121°C) for 20 min and seeded with 1.0 ml of yeast suspension (1.25 × 10^6 ^cells/ml). The flasks were incubated in a rotary shaking incubator (200 rpm) at 30°C for 48 h. A biomass ranging from 18–20 g/l was produced while 0.25% (w/v) glucose remained intact in the broth at 48 h of cultivation. Cells were harvested by centrifugation at 16,000 rpm (15,431 × g), washed free of adhering medium with ice-cold water (4°C), dried in filter paper folds (Whatman 44, Brazil) and stored at -35°C in an ultra-low freezer (Model: UF-12, Shimadzu, Tokyo, Japan).

### Biochemical reaction and critical phases

The production of L-dopa from L-tyrosine was carried out in acetate buffer (pH 3.5, 50 mM) containing (mg/ml); L-tyrosine (3.5), L-ascorbic acid (5.0) and intact cells (3.0), dispensed to a 1.25 L capacity reaction vessel (Model: 2134-nmn, Perkin Elmer, NY, USA) with a working volume of 0.75 L. Different diatomite (Sigma, St. Louis, USA) concentrations (0.5–3.0 mg/ml) were added to the reaction mixture at different time intervals (5–25 min). Reactions were carried out aerobically (1.25 l/l/min air supply, 0.5% dissolved oxygen) on a digital hot plate with magnetic stirrers (Model: G542i, Inolab, Bonn, Germany) at 50°C for different time intervals (10–60 min). The level of dissolved oxygen (DO) was measured using a Rota meter equipped with a DO-sensor (Model: RM10, Inolab, Bonn, Germany).

### Assay methods

The mixture was withdrawn from each reaction vessel, centrifuged at 9,000 rpm (8,332 × g) for 15 min and the clear supernatant was kept in the dark at ambient temperature (~20°C).

### Determination of tyrosinase activity

Tyrosinase activity was determined following a previously described method [29]. Briefly, potassium phosphate buffer (2.60 ml, 50 mM), 0.10 ml L-catechol, 0.10 ml L-ascorbic acid and 0.10 ml EDTA were mixed by inversion and equilibrated to 25°C. The ΔA_265 nm _was monitored until constant, followed by the addition of 100 μl of reaction broth. The decrease in ΔA_265 nm _was recorded for approximately 5 min. The ΔA_265 nm _was obtained using the maximum linear rate for both the test and control. Enzyme activity was determined with the following formula,

Units/mg enzyme=ΔA265 nm/min test−ΔA265 nm/min control0.001 mg enzyme/reactionmixture
 MathType@MTEF@5@5@+=feaafiart1ev1aaatCvAUfKttLearuWrP9MDH5MBPbIqV92AaeXatLxBI9gBaebbnrfifHhDYfgasaacH8akY=wiFfYdH8Gipec8Eeeu0xXdbba9frFj0=OqFfea0dXdd9vqai=hGuQ8kuc9pgc9s8qqaq=dirpe0xb9q8qiLsFr0=vr0=vr0dc8meaabaqaciaacaGaaeqabaqabeGadaaakeaacqqGvbqvcqqGUbGBcqqGPbqAcqqG0baDcqqGZbWCcqqGVaWlcqqGTbqBcqqGNbWzcqqGGaaicqqGLbqzcqqGUbGBcqqG6bGEcqqG5bqEcqqGTbqBcqqGLbqzcqGH9aqpdaWcaaqaaiabfs5aejabbgeabnaaBaaaleaacqaIYaGmcqaI2aGncqaI1aqncqqGGaaicqqGUbGBcqqGTbqBaeqaaOGaee4la8IaeeyBa0MaeeyAaKMaeeOBa4MaeeiiaaIaeeiDaqNaeeyzauMaee4CamNaeeiDaqNaeyOeI0IaeuiLdqKaeeyqae0aaSbaaSqaaiabikdaYiabiAda2iabiwda1iabbccaGiabb6gaUjabb2gaTbqabaGccqqGVaWlcqqGTbqBcqqGPbqAcqqGUbGBcqqGGaaicqqGJbWycqqGVbWBcqqGUbGBcqqG0baDcqqGYbGCcqqGVbWBcqqGSbaBaeaacqaIWaamcqGGUaGlcqaIWaamcqaIWaamcqaIXaqmcqqGGaaicqqGTbqBcqqGNbWzcqqGGaaicqqGLbqzcqqGUbGBcqqG6bGEcqqG5bqEcqqGTbqBcqqGLbqzcqqGVaWlcqqGYbGCcqqGLbqzcqqGHbqycqqGJbWycqqG0baDcqqGPbqAcqqGVbWBcqqGUbGBcqqGTbqBcqqGPbqAcqqG4baEcqqG0baDcqqG1bqDcqqGYbGCcqqGLbqzaaaaaa@960D@

### One enzyme unit

One unit of tyrosinase activity is equal to a ΔA_265 nm _of 0.001 per min at pH 6.5 at 25°C in a 3.0 ml reaction mixture containing L-catechol and L-ascorbic acid.

### Determination of L-dopa production and L-tyrosine consumption

L-dopa production and L-tyrosine consumption were determined following procedures previously described [10,30].

#### a) L-dopa

One milliliter of supernatant from the reaction mixture was added to 1.0 ml of 0.5 N HCl along with 1.0 ml of nitrite molybdate reagent (10% w/v sodium nitrite + 10% w/v sodium molybdate) (a yellow coloration appeared) followed by the addition of 1.0 ml of 1.0 N NaOH (a red coloration appeared). Total volume was brought to 5.0 ml with distilled water. Transmittance (%) was compared using a double beam UV/VIS scanning spectrophotometer (Cecil-CE 7200-series, Aquarius, London, UK) at 456 nm wavelength and the amount of L-dopa produced was determined from the standard curve.

#### b) L-tyrosine

One millilitre of the supernatant from the same reaction mixture was added to 1.0 ml of mercuric sulphate reagent (15%, w/v mercuric sulphate prepared in 5.0 N H_2_SO_4_). The test tubes were placed in a boiling water bath for 10 min and then cooled to an ambient temperature. A total of 1.0 ml of nitrite reagent (0.2% w/v sodium nitrite) was added to each tube, followed by the addition of distilled water to a final volume of 5.0 ml. Transmittance was measured by spectrophotometer (546 nm wavelength) with the amount of residual L-tyrosine determined from the tyrosine-standard curve.

### Determination of protein content

Protein in the reaction broth (with and without diatomite addition) was determined using Bradford reagent [31] with bovine serum albumin (BSA) as a standard.

### Kinetic and statistical depiction

Kinetic parameters for L-dopa production and L-tyrosine consumption were previously studied [32]. The product yield coefficient (Y_p/s_) was determined using the relationship Y_p/s _= dP/dS, while the volumetric rate for substrate utilization (Q_s_) was determined from the maximum slope in a plot of substrate utilized vs. time of biomass cultivation. Specific rate constants for substrate utilization (q_s_) were calculated by the equation i.e., qs = μ × Y_s/x_. The significance of results has been presented in the form of probability, using post-hoc multiple ranges under analysis of variance [33].

## Abbreviations

L-dopa, 3,4-dihydroxy phenyl L-alanine; rpm, revolutions per minute, EDTA, ethylene diamine tetra acetic acid; BSA, bovine serum albumin.

## Authors' contributions

SA conceived of the study; JS provided the critical review and also helped in the interpretation of results; HI helped in the funding and also gave necessary guidelines for the research work. All authors read and agreed to the final manuscript.
